# Enhancing Wheat Growth, Physiology, Yield, and Water Use Efficiency under Deficit Irrigation by Integrating Foliar Application of Salicylic Acid and Nutrients at Critical Growth Stages

**DOI:** 10.3390/plants13111490

**Published:** 2024-05-28

**Authors:** Salah El-Hendawy, Nabil Mohammed, Nasser Al-Suhaibani

**Affiliations:** Department of Plant Production, College of Food and Agriculture Sciences, King Saud University, Riyadh 11451, Saudi Arabia

**Keywords:** application timing, arid regions, grain filling stage, heading stage, heatmap, micronutrients, tillering stage

## Abstract

Transitioning from full to deficit irrigation (DI) has become a key strategy in arid regions to combat water scarcity and enhance irrigation water use efficiency (IWUE). However, implementing DI requires additional approaches to counter its negative effects on wheat production. One effective approach is the foliar application of salicylic acid (SA), micronutrients (Mic; zinc and manganese), and macronutrients (Mac; nitrogen, phosphorus, and potassium). However, there is a lack of knowledge on the optimal combinations and timing of foliar application for these components to maximize their benefits under arid conditions, which is the primary focus of this study. A two-year field study was conducted to assess the impact of the foliar application of SA alone and in combination with Mic (SA + Mic) or Mic and Mac (SA + Mic + Mac) at various critical growth stages on wheat growth, physiology, productivity, and IWUE under DI conditions. Our result demonstrated that the foliar application of different components, the timing of application, and their interaction had significant effects on all investigated wheat parameters with few exceptions. Applying different components through foliar application at multiple growth stages, such as tillering and heading or tillering, heading, and grain filling, led to significant enhancements in various wheat parameters. The improvements ranged from 7.7% to 23.2% for growth parameters, 8.7% to 24.0% for physiological traits, 1.4% to 21.0% for yield and yield components, and 14.8% to 19.0% for IWUE compared to applying the components only at the tillering stage. Plants treated with different components (SA, Mic, Mac) exhibited enhanced growth, production, and IWUE in wheat compared to untreated plants. The most effective treatment was SA + Mic, followed by SA alone and SA + Mic + Mac. The foliar application of SA, SA + Mic, and SA + Mic + Mac improved growth parameters by 1.2–50.8%, 2.7–54.6%, and 2.5–43.9%, respectively. Yield parameters were also enhanced by 1.3–33.0%, 2.4–37.2%, and 3.0–26.6% while IWUE increased by 28.6%, 33.0%, and 18.5% compared to untreated plants. A heatmap analysis revealed that the foliar application of SA + Mic at multiple growth stages resulted in the highest values for all parameters, followed by SA alone and SA + Mic + Mac applications at multiple growth stages. The lowest values were observed in untreated plants and with the foliar application of different components only at the tillering stage. Thus, this study suggested that the foliar application of SA + Mic at various growth stages can help sustain wheat production in arid regions with limited water resources.

## 1. Introduction

By 2030, the global demand for fresh water is projected to exceed supply by 40%, emphasizing the need for sustainable water management practices, especially in agriculture. Agriculture is the largest consumer of fresh water, comprising 75–80% of total water usage in many countries. Most importantly, countries relying on non-renewable fossil water are facing more frequent water shortages due to climate change, sparse rainfall, high temperatures, population growth, urbanization, and industrialization. Unfortunately, this problem is worsening year by year in arid and semiarid countries. As a result, many governments in these countries, such as Saudi Arabia, have implemented several regulations to reduce water allocation to the agricultural sector and conserve groundwater for upcoming generations, such as installing water meters on pumping stations and implementing pricing for irrigation water [1,2]. These regulations have forced many agricultural companies to switch from full irrigation to deficit irrigation (DI), which means supplying water to crops at levels below their full water requirements. This is because the increased yield from full irrigation did not compensate for the costs of this extra irrigation water [3,4]. However, growing plants under DI conditions has consequences on several morphological, physiological, and biochemical aspects of plants, ultimately leading to a significant reduction in final grain yield (GY). In wheat crops, this reduction can exceed 50% [5,6,7,8]. Therefore, there is an urgent need to apply effective strategies to mitigate the negative effects of DI on the growth and production of wheat crop. 

Due to the close relationship between nutrient and water uptake, plants are unable to effectively uptake, transport, and metabolize many macronutrients (Mac) and micronutrients (Mic) under DI conditions. Studies have shown a decreased uptake and translocation of essential Mac, such as N, P, and K, in different plant crops under DI conditions [9,10,11,12]. For example, Bista et al. [13] found that subjecting drought-sensitive barley and corn genotypes to DI conditions led to a significant decrease in N and P concentrations by 44–51% and 39–48%, respectively. A reduced amount of K was also observed in several crops that were subjected to DI [14]. Under deficit irrigation, Mic deficiencies may occur, as they are converted to less soluble forms. Conversely, in moist soil conditions, Mic are converted to more soluble and reduced forms [10,15]. Generally, the decrease in Mac and Mic uptake under DI conditions may be due to reduced water content in plants, leading to stomatal closure and decreased transpiration rates. This limits nutrient uptake by roots and their transport to shoots. Additionally, low soil moisture reduces nutrient diffusion and mass flow in the soil, inhibits root growth, and decreases nutrient inflow per unit of root length and biomass. Furthermore, low soil moisture reduces nutrient uptake by decreasing nutrient mineralization and inhibiting the active transport and membrane permeability of cations, which in turn leads to the decreased absorption of cations through the roots [16,17,18,19]. Therefore, it can be concluded that the reduced uptake and transport of essential nutrients in plants is a common issue under water-deficient situations. Due to the significant roles of Mac and Mic nutrients in mitigating the negative effects of drought stress on crop growth and production, through their involvement in various physiological and biochemical processes in plants, their foliar application could be a practical and cost-effective method to improve wheat performance under DI conditions. Previous studies have demonstrated that applying Mac and/or Mic nutrients to the leaves can improve crop growth and yield in water-deficient conditions [20,21,22,23,24]. However, most of these studies were conducted in controlled pot experiments, and there is limited research to support the use of foliar nutrient application to reduce DI in field conditions under arid climates. This study aims to address this gap in the literature.

When plants experience water deficit stress conditions, it disrupts several physiological and biochemical processes, including photosynthesis, biomass accumulation, hormonal and nutrient balance, chlorophyll biosynthesis, and water relations. This ultimately leads to a significant reduction in crop growth and yield [7,8,25]. Several plants respond to water deficit stress by producing some important signaling molecules, such as salicylic acid (SA). SA acts as a phytohormone and helps plants adapt to various abiotic stressors. SA plays a crucial role in regulating important physiological and metabolic processes in plants, such as photosynthesis, transpiration, cell growth, plant development, water uptake, ion transport, osmotic adjustments, stomatal opening, and defense mechanisms [26,27,28]. Therefore, applying SA externally could be a practical and cost-effective approach to enhance wheat growth and production under DI conditions. Numerous studies have confirmed the positive impacts of the exogenously foliar application of SA on the growth and production attributes of different field crops under water deficit stress. For example, the exogenous application of SA improves the growth and yield attributes of different cereal crops under water deficit conditions by increasing relative water content, photosynthetic pigments and activities, stomatal conductance, water and nutrient use efficiency, and antioxidant capacity [29,30,31,32,33,34]. 

The foliar application of Mic and Mac can help reduce the negative effects of water deficit stress. Additionally, the exogenous application of SA can alleviate drought stress and enhance defense mechanisms against it. Therefore, combining SA, Mic, and Mac applications may be more effective in improving plant growth and productivity compared to applying each one alone. For example, spraying wheat plants with SA and zinc reduced the negative effects of water deficiency, leading to increased chlorophyll content, relative water content, plant height, spike length, number of grains per spike, 1000-grain weight, grain yield, and water use efficiency [35]. Likewise, Alotaibi et al. [36] concluded that combining micronutrients with SA is more effective in enhancing the growth, yield parameters, and irrigation water use efficiency (IWUE) of wheat plants under water deficit compared to using these compounds individually. Safar-Noori et al. [37] found that combining SA with a sufficient amount of K enhanced wheat growth and production under drought stress more effectively than applying them separately. Therefore, in this study, we hypothesize that combining foliar applications of SA and plant nutrients can effectively mitigate the effects of drought-induced stress in wheat compared to applying each treatment separately. This integrated approach may have synergistic effects in alleviating the negative impacts of drought stress on the various morpho-agro-physiological traits of wheat. 

In wheat, several growth stages are sensitive to water deficit stress. Early water deficit stress can reduce the number of plants and tillers per unit area, ultimately leading to a lower yield potential, including a decrease in the number of spikes per square meter [38,39]. Water deficit stress during the stem elongation stage has a negative impact on fertility and floret formation, ultimately leading to a decrease in the number of grains per spike. Additionally, post-anthesis water deficit stress reduces the rate and duration of grain filling, resulting in a decrease in 1000-grain weight [40]. The final grain yield is determined by two major components: grain number per unit area and grain weight. The grain number per unit area is determined during the pre-anthesis stage, while the grain weight is determined at the post-anthesis stage. Therefore, water deficit stress at both stages can lead to yield losses of up to 69% [7]. Research has shown that the number of plants per unit area, the number of spikes per plant, and the number of grains per spike contribute 20%, 29%, and 51%, respectively, to the grain number per unit area [41,42]. This also reflects that water deficit stress at the pre-anthesis stage can result in a significant reduction in the final yield of wheat. To reduce the negative effects of water deficit stress on wheat, it is important to target the most sensitive growth stages. As mentioned earlier, the foliar application of SA, Mac, and Mic can effectively mitigate these effects. Therefore, with exogenous applications of these components and by targeting the most water-sensitive growth stages, the negative impacts of DI stress on wheat growth and production can be effectively minimized. However, previous studies have shown that although water deficit stress affects the growth and productivity of wheat at different growth stages, the efficiency of the exogenous application of these components in alleviating this stress varies with different growth stages [19,43]. For instance, Ning et al. [43] reported that the foliar application of silicon (Si) to wheat crops during the reproductive stage was more effective in mitigating the adverse effects of drought stress on physiological traits and grain yield as opposed to applying it during the jointing stage. Gong et al. [44] found that the foliar application of Si is more effective in enhancing wheat defense against oxidative stress during drought when applied at the grain filling stage, with minimal impact when applied at the booting stage. However, in other studies, it was reported that the booting to anthesis growth stages in wheat are optimal for the foliar application of micronutrients (such as Zn, Mn, and B) to enhance wheat production under drought stress, especially when drought occurs during the late growth stage [45,46]. Khan et al. [47] also found that the foliar application of micronutrients at multiple growth stages, from tillering to heading, significantly enhanced the growth and yield of wheat under drought stress. Therefore, in this study, we assume that when the foliar application of SA and/or plant nutrients coincides with the growth stages most sensitive to water stress, it will help overcome the negative impacts of DI stress efficiently.

In this context, the main objective of this study was to investigate how the foliar application of different combinations of SA, Mic, and Mac at various sensitive growth stages of wheat affects its growth, yield, and irrigation water use efficiency (IWUE). The goal was to find the most effective combinations and optimal timing for application to improve wheat production under DI conditions.

## 2. Results

### 2.1. Growth Parameters

Table 1 shows the analysis of variance (ANOVA) for the effects of seasons (S), application time (AT), foliar application treatments (F), and their possible interaction on different growth parameters measured at 95 and 115 days after sowing (DAS). The effect of S and its interaction with F on all growth parameters was not significant. The interaction with AT was also not significant except for the green leaf number (GLN) at 95 DAS and the tiller number (TN) at 115 DAS. The AT treatment had a significant effect on all parameters except plant height (PH) at 95 and 115 DAS and TN at 95 DAS. Similarly, the F treatment had a significant effect on all parameters except TN at 95 DAS. 

The results in Table 1 show that applying different components at both the tillering and heading stages (T + H), as well as the tillering, heading, and grain filling stages (T + H + G), resulted in enhanced growth parameters at 95 and DAS compared to applying them only at the tillering stage (T). On average over two seasons, applying components at T + H and T + H + G resulted in improvements in growth parameters by 13.5–18.7% and 12.7–19.2% at 95 DAS and 7.7–18.6% and 9.2–23.2% at 115 DAS, respectively, compared to applying them at the stage (Table 1). Regardless of the application time, the foliar application of different components significantly improved all growth parameters except TN at 95 DAS compared to the control treatment. The highest values for all growth parameters were observed with the foliar application of SA and Mic (SA + Mic), followed by SA, Mic, and Mac (SA + Mic + Mac) or SA alone. On average over two seasons, the foliar application of SA, SA + Mic, and SA + Mic + Mac improved various growth parameters by 1.2–28.6%, 2.7–31.8%, and 2.5–24.6% at 95 DAS, and 3.5–50.8%, 4.1–54.6%, and 4.6–43.9% at 115 DAS, respectively, compared to the control treatment (Table 1).

**Figure 1:**
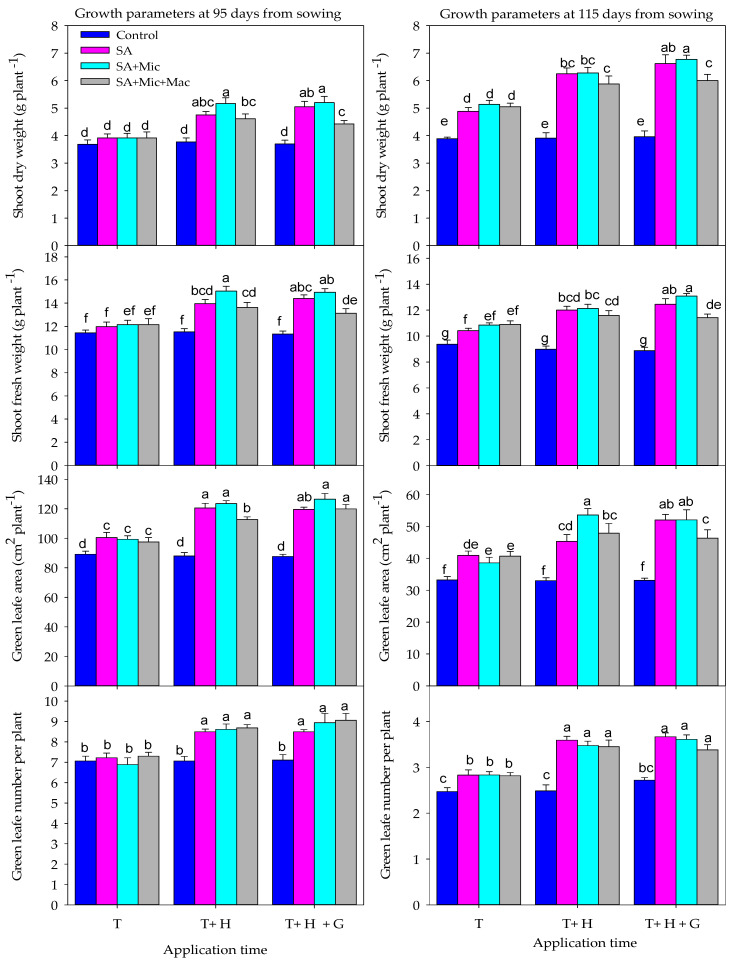
The combined effect of the application timing and the foliar application of salicylic acid (SA), macronutrients (Mac), and micronutrients (Mic) on different growth parameters over two growing seasons. T, T + H, and T + H + G indicate foliar application at tillering, tillering and heading, and tillering, heading, and grain filling growth stages, respectively. Values are the means of three replications (n = 3). Bars with the same letter are not significantly different at the 0.05 level based on Tukey’s test.

The combined effect of AT and F on different growth parameters reveals that applying the different components at the T stage only resulted in a slight improvement compared to the control treatment. However, a significant improvement was observed when the components were applied at multiple growth stages (T + H and T + H + G). The highest growth parameters were observed with the foliar application of SA+Mic and SA alone at the T + H + G stages, followed by SA + Mic at the T + H stages. SA alone at the T + H stages showed comparable values for growth parameters to SA + Mic + Mac at the T + H + G stages (Figure 1).

### 2.2. Physiological Parameters

The ANOVA analysis showed that AT, F, and their interaction had a significant effect (*p* ≤ 0.001) on relative water content (RWC) and different chlorophyll (Chl) contents at 95 and 115 DAS. However, S had a significant effect (*p* ≤ 0.05) only on total Chl contents (Chlt) at 115 DAS (Table 2). The interaction between S and AT had a significant effect on RWC at 95 DAS but not at 115 DAS; the opposite was true for different Chl contents. The interaction between S and F significantly affected the RWC and different Chl levels at 95 DAS and only Chl-b at 115 DAS. The three-way interaction between AT, F, and S did not significantly affect the RWC and Chl contents at 95 or 115 DAS (Table 2). 

**Figure 2:**
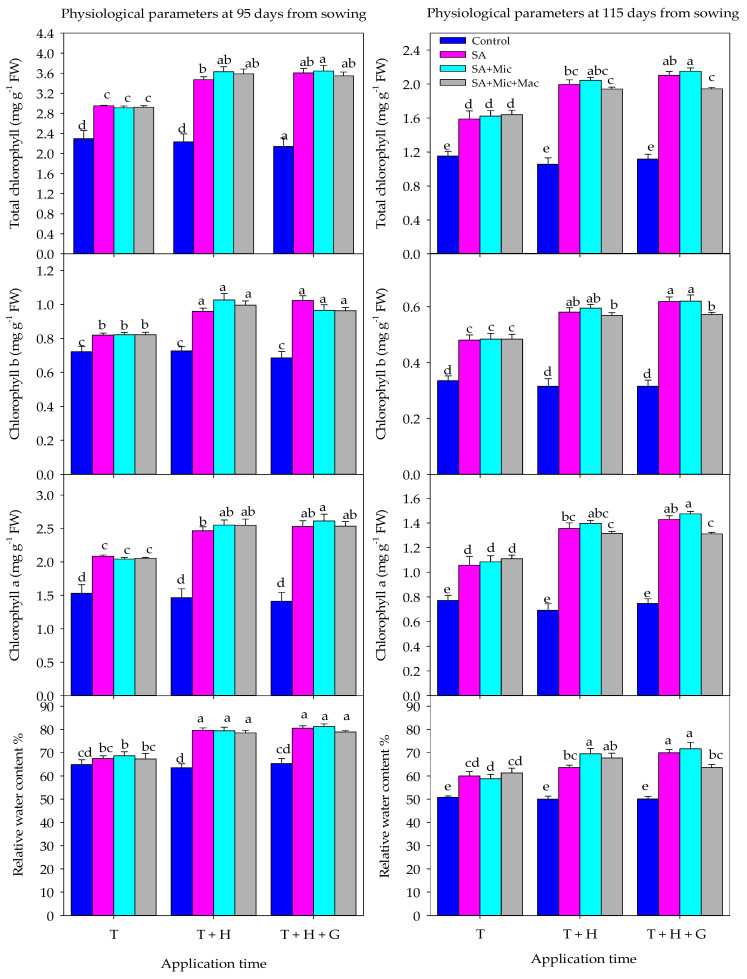
Interactive effect of application timing (AT) and foliar treatments of salicylic acid (SA), macronutrients (Mac), and micronutrients (Mic) on different physiological parameters over two growing seasons. T, T + H, and T + H + G indicate foliar application at tillering, tillering and heading, and tillering, heading, and grain filling growth stages, respectively. Values are the means of three replications (n = 3). Bars sharing the same letter are not significantly different at the 0.05 level based on Tukey’s test.

The results in Table 2 show that the effectiveness of the foliar application of different components in improving RWC and Chl contents under DI conditions depends on the timing of their application. Applying them at multiple growth stages is more effective than applying them at only a single stage. The foliar application of different components at T + H or T + H + G growth stages enhanced RWC and Chl contents by 12.1–17.7% and 14.0–18.2% at 95 DAS and by 8.7–19.0% and 10.6–24.0% at 115 DAS, respectively, compared to applying them only at the T stage (Table 2). Regardless of AT, the foliar application of SA alone or in combination with Mic and/or Mac had a positive effect on the RWC and different Chl contents under DI conditions. The foliar application of SA, SA + Mic, and SA + Mic + Mac significantly enhanced RWC, Chla, Chlb, and Chlt by 15.9–18.3%, 60.5–63.3%, 31.0–32.4%, and 50.5–52.7% at 95 DAS and by 27.6–32.5%, 68.9–78.4%, 68.8–75.1%, and 67.3–76.4% at 115 DAS, respectively, compared to the non-treated treatment (Table 2). Additionally, the effects of different components on RWC and Chl contents also depend on the AT. In general, RWC and Chl contents were significantly improved by the foliar application of different components at T + H and T + H + G growth stages compared to foliar application at the T stage alone (Figure 2). 

When applied at the T stage, there was no significant difference between the foliar application of SA, SA + Mic, and SA + Mic + Mac. However, when applied at T + H or T + H + G, the highest values for RWC and different Chl contents were obtained with the foliar application of SA + Mic at T + H and T + H + G, followed by the foliar application of SA alone at T + H + G and SA + Mic + Mac at the T + H growth stage (Figure 2). 

### 2.3. Yield Parameters and Irrigation Water Use Efficiency

The F-test from the ANOVA showed that AT, F, and their interaction had a significant effect (*p* ≤ 0.05 and 0.01) on all yield parameters and irrigation water use efficiency (IWUE) except for spike length (SL) and biological yield (BY). The F did not have a significant effect on SL, and the interaction between AT and F did not have a significant effect on SL and BY (Table 3). The S and their interaction with AT and F as well as the three-way interaction (F × AT × S) did not have a significant effect on all yield parameters and IWUE except for BY, which was affected by S (Table 3). 

The results in Table 3 show that applying a foliar treatment at multiple growth stages is more effective in improving yield parameters and IWUE compared to applying it only at one early growth stage. Foliar application at T + H and T + H + G increased the spike length (SL) by 1.4% and 3.5%, the grain weight per spike (GWPS) by 13.3% and 21.0%, the grain number per spike (GNPS) by 5.7% and 11.6%, the thousand grain weight (TGW) by 6.3% and 7.2%, the grain yield per ha (GY) by 14.7% and 18.7%, the biological yield per ha (BY) by 8.0% and 10.0%, the harvest index (HI) by 5.8% and 7.5%, and IWUE by 14.8% and 19.0%, respectively, compared to foliar application at only the T stage. The results in Table 3 also show that the foliar application of SA, either alone or in combination with Mic.

Mac increased yield parameters and IWUE compared to untreated plants. The highest values for yield parameters and IWUE were observed when plants were treated with a combination of SA and Mic, followed by plants treated with SA alone. Plants treated with SA, SA + Mic, and SA + Mic + Mac showed increases in several yield parameters and IWUE compared to untreated plants. Specifically, they had higher SL by 1.3%, 2.4%, and 3.0%; greater GWPS by 33.0%, 37.2%, and 26.6%; increased GNPS by 11.9%, 13.1%, and 9.7%; higher TGW by 17.5%, 20.1%, and 14.4%; greater GY by 28.9%, 33.2%, and 18.9%; higher BY by 13.1%, 13.8%, and 13.3%; increased HI by 13.2%, 16.5%, and 4.7%; and improved IWUE by 28.6%, 33.0%, and 18.5%, respectively.

**Figure 3:**
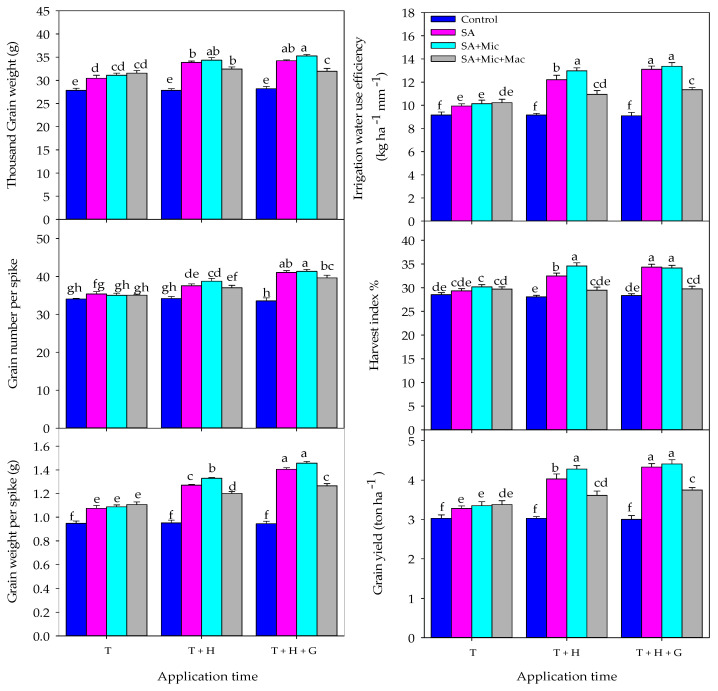
The interactive effect of application timing and the foliar treatment of salicylic acid (SA), macronutrients (Mac), and micronutrients (Mic) on different yield, yield components, and irrigation water use efficiency over two growing seasons. T, T + H, and T + H + G indicate foliar application at tillering, tillering and heading, and tillering, heading, and grain filling growth stages, respectively. Values are the means of three replications (n = 3). Bars sharing the same letter are not significantly different at the 0.05 level based on Tukey’s test.

Figure 3 shows the impact of the interaction between AT and F on yield parameters and IWUE. In general, applying SA alone or in combination with Mic and/or Mac at the T stage did not effectively improve yield parameters and IWUE only compared to applications at multiple stages (T + H and T + H + G). The highest values for yield parameters and IWUE were achieved when SA was sprayed alone or in combination with Mic at the T + H + G growth stage, followed by the application of SA + Mic at the T + H growth stage. The application of SA alone at the T + H growth stage was more effective in improving yield parameters and IWUE compared to the combined application of SA with Mic and Mac at either the T + H or T + H + G growth stages (Figure 3). 

### 2.4. Heatmap Clustering Analysis

**Figure 4:**
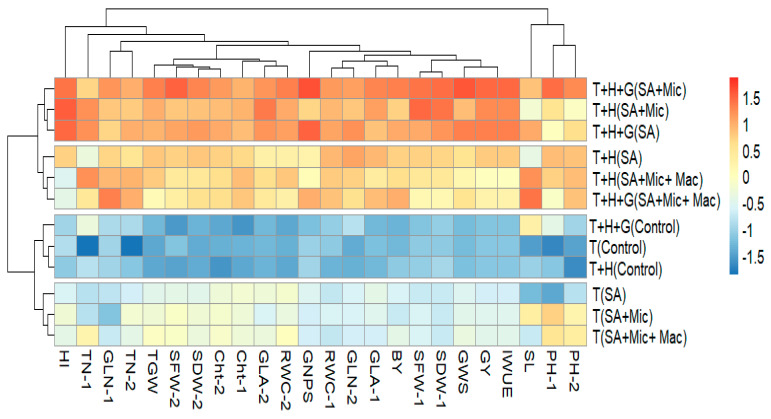
The heatmap of the different studied parameters of wheat and the various of application timing (AT) and foliar treatments (F) of salicylic acid (SA), macronutrients (Mac), and micronutrients (Mic) under limited irrigation. T, T + H, and T + H + G indicate foliar application at tillering, tillering and heading, and tillering, heading, and grain filling growth stages, respectively. PH, plant height (cm plant^−1^); TN, tiller number per plant; GLN, green leaf number per plant; GLA, green leaf area (cm^2^ plant^−1^); SFW, shoot fresh weight (g plant^−1^); SDW, shoot dry weight (g plant^−1^); RWC, relative water content (%); Chlt, total chlorophyll content (mg g^−1^ fresh weight), SL, spike length (cm); GNPS, grain number per spike; GWS, grain weight per spike (g); TGW, thousand-grain weight (g); GY, grain yield (ton ha^−1^); BY, biological yield (ton ha^−1^); HI, harvest index (%); IWUE, irrigation water use efficiency (kg mm^−1^ ha^−1^). The numbers 1 and 2 indicate traits measured at 95 and 115 days from sowing, respectively.

The heatmap clustering analysis (HCA) was performed using 24 parameters and 12 treatments, which included various combinations of AT and F (Figure 4). The HCA divided the 12 treatments into four groups. The foliar application of SA alone at the T + H + G growth stage or in combination with Mic applied at either T + H or T + H + G were grouped together and produced the highest values for almost all 24 parameters. The treatments involving the foliar application of SA, either alone or in combination with Mic and Mac at the T stage only, were grouped together and resulted in the lowest values for all parameters. The three untreated treatments (control) were grouped together and showed a negative correlation with all the studied parameters. HCA indicated that applying SA with both Mic and Mac at multiple growth stages improved various agro-physiological and yield parameters as well as IWUE. However, these treatments were not as effective as applying SA alone or in combination with Mic alone at multiple growth stages (Figure 4).

## 3. Discussion

Transitioning from full to deficit irrigation (DI) has become an important strategy in arid and semiarid countries to conserve non-renewable groundwater. However, exposing crops to DI conditions can reduce their production by over 50%, as observed in wheat crop [6,7,8,42]. Most importantly, water deficit can also cause nutrient deficiencies in plants by reducing the availability, uptake, translocation, and metabolism of essential Mic and Mac nutrients [19,48,49]. This indicates that when water deficit and nutrient deficiency stress occur together, it can significantly decrease wheat yield under DI conditions. A study by Bagci et al. [50] found that drought stress significantly reduced wheat crop yield in zinc-deficient plants. Therefore, several studies have suggested that balanced plant mineral nutrients are crucial for improving crop growth and productivity and mitigating the negative effects of water deficit stress in a sustainable manner [9,51,52]. Additionally, the foliar application of compatible solutes, such as SA, can also play a crucial role in mitigating the negative impacts of water deficit stress on various physiological and biochemical processes in plants [53,54,55]. Therefore, the exogenous application of SA and essential plant nutrients can be a cost-effective way to improve the growth and production of wheat under DI conditions. However, the effectiveness of this approach depends on several factors, such as the concentration, timing, and method of application as well as any synergistic effects [56,57,58,59]. Therefore, this study aims to examine the impact of applying SA alone and in combination with plant nutrients during sensitive growth stages of wheat on agro-physiological and yield parameters as well as IWUE under DI conditions. 

Previous studies have demonstrated that water deficit stress has a significant impact on different growth stages of wheat plants, including tillering, stem elongation, heading, anthesis, and grain filling. The yield components that contribute to the final grain yield (GY) vary at each growth stage, and the severity of the water deficit stress depends on how these components are impacted [60,61,62,63,64]. The final GY of wheat is determined by the number of tillers and spikes per plant, the number of spikelets per spike, and the number and weight of grains per spike [61,62]. The spike number per plant is closely associated with the tillering capacity. Additionally, water deficit and nutrient deficiency during the tillering stage can result in more aborted or infertile tillers, reducing the total number of spikes per plant [64,65,66]. Importantly, spikelet initiation begins in the seedling stage and continues into the tillering stage, while floret initiation starts during tillering and extends into the stem elongation stages [62]. Therefore, water deficit stress during the early growth stages of wheat (tillering and stem elongation) can significantly reduce the number of spikelets and spikes per plant, ultimately affecting the final GY. Thus, it is essential to mitigate the negative impacts of water deficit stress in wheat during the vegetative stage to optimize GY. Therefore, in this study, we included tillering growth stages in all treatments to evaluate the impact of different application timings of various components. Water deficit stress during pre-anthesis stress can decrease the number of spikelets per spike. Post-anthesis and grain filling stress can lead to a reduction in the number and weight of grains [60,67,68,69,70]. 

The above evidence indicates the exogenous application of SA and plant nutrients can help mitigate the negative effects of water deficit stress, particularly when applied during the most sensitive growth stages. The results of this study indicate that the exogenous application of SA, Mic, and Mac at multiple growth stages was more effective in improving wheat growth, physiology, yield, and IWUE compared to applying them at a single growth stage (Table 1, Table 2 and Table 3 and Figure 1, Figure 2 and Figure 3). Additionally, the foliar application of these components at the late growth stage (heading and grain filling) was more effective than applying them at the early growth stage (tillering) (Table 1, Table 2 and Table 3 and Figure 1, Figure 2 and Figure 3). Because water deficit stress during anthesis and grain filling stages has the greatest impact on yield loss, this may explain why the foliar application of different components is more effective when applied at heading and grain filling stages. It is important to note that the anthesis and grain filling stages are non-compensatory growth stages in wheat crops. This means that any yield components determined around both stages, such as grain number and weight, will be significantly affected by water deficit stress. This also confirms the importance of the foliar application of SA and plant nutrients at the heading and grain filling stages in this study in mitigating the negative impacts of DI conditions. Similarly, previous research has shown that the most effective method to enhance crop production under water deficit conditions is by applying SA and plant nutrients to the leaves during the late-season growth stages, particularly from heading to grain filling stages. Foliar application during these stages has been shown to have a positive impact on grain yield and its components, such as GWPS, GNPS, and TGW. In contrast, foliar application during the tillering stage did not have a significant effect on final grain yield [27,46,49,71,72]. Karim et al. [49] also found that applying micronutrients (Mn, B, and Zn) to cereal crops during the booting to grain filling stage helped to mitigate yield loss caused by drought stress. Therefore, we can conclude that the timing of applying SA and plant nutrition is crucial for improving wheat production under DI conditions. Applying these components multiple times during the late growth stage effectively mitigates the negative impacts of water deficit stress on wheat growth, physiology, production, and water use efficiency (Table 1, Table 2 and Table 3 and Figure 1, Figure 2 and Figure 3). The effectiveness of the foliar application of SA and/or Mic and Mac at the late growth stage in enhancing the growth and production of wheat under DI conditions may be attributed to several factors. First, the foliar application of these components during heading and grain filling stages helps the flag leaf survive longer and delays early leaf senescence. This leads to better grain numbers, grain weight, and TGW, which are main components for final grain yield [73,74,75]. Second, the foliar application of these components at later stages facilitates the remobilization of assimilates from the leaves and stems to the grains, resulting in increased grain weight and TGW [76]. Third, the foliar application of these components at later stages improves the duration and rate of grain filling, leading to an increase in grain weight and TGW. Fourth, due to the important role of Mic nutrients, such as zinc and boron, in pollen viability and grain setting, the foliar application of these nutrients at later stages increases the number and weight of grains per spike [77]. Lastly, the foliar application of Mic nutrients at later stages improves the development of the fine root system, resulting in improved water use efficiency and crop yield [12,49]. 

In this study, it was also found that the foliar application of SA alone or in combination with Mic and/or Mac improved the growth, yield, and IWUE of wheat under DI conditions compared to untreated plants. Furthermore, the use of SA alone or in combination with Mic was more effective than the combination of SA with both Mic and Mac nutrients (Table 1, Table 2 and Table 3 and Figure 1, Figure 2 and Figure 3). Treating plants with these components under water deficit stress has a positive effect because they play a central role in regulating several physiological and biochemical processes within the plant. For example, SA is known to regulate various physiological processes in plants, such as water uptake, transpiration, photosynthesis, stomatal conductance, cell membrane stability, plant pigments, hormone synthesis, reactive oxygen species (ROS) scavenging, osmolyte accumulation, and ion and photosynthetic product translocation [78,79,80,81]. It also plays a crucial role in improving plant water status and water use efficiency by increasing leaf diffusive resistance and reducing transpiration rates [82]. Additionally, the application of SA has been found to promote cell division and enlargement, leading to improved growth, development, and ultimately yield-related parameters under water deficit stress [31,83]. Hafez [84] also noted that the foliar application of SA increased the flow of metabolites to developing grains, resulting in a higher number of grains per spike and higher TGW. These various important functions of SA may explain why its foliar application alone improved the growth, yield, and IWUE of wheat under DI conditions in this study.

It is interesting to note that several Mic nutrients are involved in various physiological processes related to tolerance to water deficit stress. For instance, zinc (Zn), boron (B), and manganese (Mn) are essential for maintaining photosynthetic activity and the function of enzymes that are sensitive to water deficit stress, as well as for preserving membrane integrity [10,51,85]. Additionally, they are crucial for protecting plants against ROS produced during abiotic stress [86]. They also play a prominent role in cell division, metabolism, carbohydrate transport, ion absorption, water relations, growth hormone synthesis, pollen viability, and enzyme activity [10,49,85,87]. Water deficit stress during the anthesis growth stage can harm the development of male reproductive organs in cereal crops and reduce the translocation of assimilates from shoots to spikes as well as pollen viability. However, these negative effects can be lessened by boosting levels of micronutrients, such as Mn, B, and Zn, in the plants at this stage [49]. Previous studies have also shown that the foliar application of Mic nutrients under water deficit stress not only prevents nutrient deficiency in plant tissue but is also important in helping plants to absorb water and enhance WUE by promoting root system development and root hair growth [24,88,89]. All aforementioned facts about the important role of Mic nutrients in mitigating the negative impact of water deficit stress may explain why the foliar application of Mic nutrients with SA improved the growth, yield, and IWUE of wheat under DI conditions in this study. 

Interestingly, this study showed that combining Mac nutrients (N, P, and K) with SA and Mic nutrients (SA + Mic + Mac) improved wheat growth, production, and IWUE under DI conditions but not as much as treatments with only SA or a combination of SA and Mic nutrients. These results indicate that Mac nutrients are less effective than SA and Mic in enhancing wheat growth, production, and water use efficiency under DI conditions. The limited effectiveness of the foliar application of Mac nutrients under DI conditions may be due to their promotion of vegetative growth and leaf area, which increases water loss through transpiration. Additionally, some mineral nutrients, such as P and Zn, can have significant antagonistic effects, meaning that the ability of one nutrient to mitigate the negative effects of water deficit stress may be diminished by the presence of the other [90]. Furthermore, Dass et al. [85] found that hormonal changes or loss of anabolic enzymes can prevent the effective use of the foliar application of nitrogen. They also observed that the foliar application of urea can cause leaf burn (leaf tip necrosis) and result in a significant decrease in yield. Prasertsak and Fukai [91] found that the application of nitrogen to rice crops resulted in rapid water deficit stress. This was evidenced by a decrease in leaf water potential and a high rate of leaf mortality during the stress period. However, other studies found that applying Mac nutrients, such as N, P, Ca, and K, through foliar application alone can enhance the yield and yield components of various field crops when they are subjected to drought stress [52,92,93,94]. These differences in the effectiveness of the foliar application of Mac nutrients in alleviating water deficit stress may be attributed to variations in climate conditions and crop types among studies. Additionally, most research on this topic has been conducted in controlled pot experiments, with limited evidence supporting the use of foliar nutrient application to alleviate water deficit stress in field conditions. Therefore, the recommendation to use foliar nutrient application to reduce water deficit stress should be based on specific experimental conditions.

## 4. Materials and Methods

### 4.1. Experimental Site Description and Cultivation Conditions

**Figure 5:**
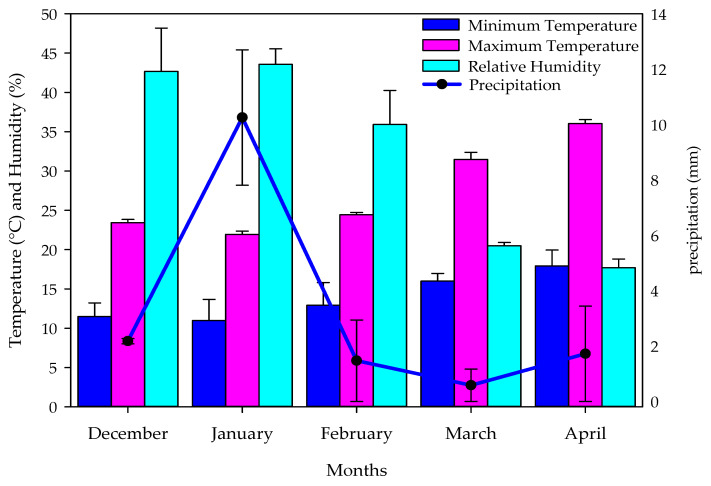
Different meteorological data of the experimental site over two wheat growing seasons.

Two field experiments were carried out during the winter seasons from December to April in 2020/2021 and 2021/2022 at the Agricultural Research Farm of the Plant Production Department, College of Food and Agriculture Sciences, King Saud University, Riyadh, Saudi Arabia (24°25′03″ N 46°39′17″ E), 570 m above sea level. The soil of the research farm is sandy loam texture and consist of 57.92% sand, 28.65% silt, and 13.42% clay. The experimental site has arid climatic conditions, with an annual precipitation rate of about 30 mm and low humidity. Figure 5 shows the monthly average meteorological data for temperature, relative humidity, and precipitation at the experimental site over two winter seasons.

The spring wheat variety Summit was hand-sown on December 8 in the first season and December 1 in the second season. The seeds, uniform in size and plump, were sown at a rate of 150 kg ha^−1^ in eight rows, each 4 m long and 20 cm apart. Before sowing, the soil was plowed twice, leveled, and divided into three plots, each of which represented a repetition. Each replicate was divided into three main plots (8.8 m × 4 m), and each main plot was divided into four subplots (1.6 m × 4 m) with a buffer zone of 80 cm between the two adjacent subplots. Phosphorus (P), potassium (K), and nitrogen (N) fertilizers were applied in the form of calcium superphosphate (17% P_2_O_5_), potassium sulfate (50% K_2_O), and urea (46.5% N), respectively. All P (60 kg P_2_O_5_ ha^−1^) was applied at sowing time, while K (60 kg K_2_O ha^−1^) was applied in two equal doses at the seedling and stem elongation stages. Nitrogen fertilizer was applied in three equal doses at seedling, stem elongation, and booting stages at a rate of 180 kg N ha^−1^. The crop was managed with regard to pest and weed control as needed and at the appropriate time.

Effects of different treatments of this study on growth, yield, and physiological traits of wheat were evaluated under deficit irrigation (DI). The DI was calculated based on 50% of estimated crop evapotranspiration (ETc) of wheat crop. Daily ETc was calculated by multiplying the daily reference evapotranspiration rate (ETo) by the crop coefficient (Kc) of wheat crop. We used the modified Penman–Monteith equation and daily weather data uploaded from a nearby station to calculate the daily ETo. The Kc values for the main growth stages of spring wheat from FAO-56 [95] were modified to fit the climate of the experimental site. According to this calculation, the total irrigation volume applied for DI was 3250 and 3300 m^3^ ha^−1^ in the first and second seasons, respectively. The irrigation was scheduled when the available soil water level for the plants dropped to 30%. Irrigation water was applied using a low-pressure surface irrigation system designed for small plots.

### 4.2. Experimental Design and Treatments

The study used a randomized complete block design with a split-plot arrangement and three replications. The main plot included three foliar application timings: tillering (T), tillering and heading (T + H), and tillering, heading, and grain filling (T + H + G) stages. The subplots consisted of three foliar components: salicylic acid (SA), macronutrients (N-P-K), and micronutrients (Zn and Mn). These components were combined to create different treatments: control (foliar pure water), SA foliar application, SA + micronutrient (Mic) foliar application, and SA + Mic + macronutrient (Mac) foliar application. Foliar application of SA was applied at a concentration of 2.0 mM as HOC_6_H_4_COOH. The Mic (Zn and Mn) were applied as ZnSO_4_.7H_2_O and MnSO_4_.3H_2_O at concentrations of 1.0% and 0.5%, respectively. The three Mac were applied at a concentration of 1.0%. Salicylic acid was dissolved in absolute ethanol, while the other components were dissolved in distilled water to make a stock. Then, the appropriate quantities of this stock were gently added to the distilled water to make spray solutions with the required concentrations. The various prepared solutions, each with 0.1% Tween-20, were sprayed directly onto the plant leaves until they were completely covered and the solutions ran off leaves. The different solutions were sprayed using back-mounted pressurized sprayer (16 L) with a T-jet nozzle. The sprayer was calibrated to deliver 15 mL s^−1^ at a pressure of 207 kPa.

### 4.3. Data Recorded

#### 4.3.1. Growth Parameters

At 95 and 115 days after sowing, we randomly selected ten plants from each subplot to measure PH, TN, PFW, and GLN. Then, we used an area meter (LI 3100; LI-COR Inc., Lincoln, NE, USA) to measure the leaf area per plant. To determine the PDW, we oven-dried all components of the ten plants at 80 °C until a constant weight was achieved.

#### 4.3.2. Physiological Parameters

Five leaves from the upper plant’s second leaf were randomly selected, and their fresh weight (FW) was immediately measured. The leaves were then soaked in distilled water for 24 h in the dark, and their turgor weight (TW) was determined after removing surface water. The leaves were dried at 80 °C for 72 h to obtain their dry weight (DW). The relative water content of the leaves (RWC) was calculated using the following formula:

RWC = (FW − DW)/(TW − DW) × 100(1)

Chlorophyll pigments (Chl a, Chl b, and Chlt) were quantified using the method outlined by Arnon [96] and Lichtenthaler [97]. Fresh leaf samples (0.5 g) were immersed in 80% acetone in the dark until colorless. The extract was then centrifuged, and the absorbance was measured at 645 and 663 nm using a spectrophotometer (UV-2550, Shimadzu, Tokyo, Japan). The concentrations of Chl a, Chl b, and Chlt were calculated in mg g^−1^ FW using the following equations:

Chl a = [(12.7 × A663) − (2.69 × A645)] × V/1000 × FW(2)

Chl b = [(22.9 × A645) − (4.68 × A663)] × V/1000 × FW.(3)

Chl t = [(20.2 × A645) + (8.02 × A663)] × V/1000 × FW (4)

where A is the absorbance at specific wavelengths, V is the final volume of extract (ml), and FW is the fresh weight of tissue extracted (g).

#### 4.3.3. Yield and Yield Components

Fifty spikes were randomly selected from each subplot to measure SL, GWS, NGPS, and TGW at maturity in mid-April for both seasons. The inner five rows of each subplot (3 m^2^) were manually harvested, sun-dried, and weighed to determine BY in kg per 3.0 m^2^, converted to ton ha^−1^. The spikes of harvested area were threshed, and grains were collected, cleaned, dried, and weighed to determine GY in kg per 3.0 m^2^, also converted to ton ha^−1^. Harvest index (HI) was calculated as GY divided by BY, while IWUE was calculated as GY divided by ETc.

### 4.4. Data Analysis

Analysis of variance was conducted for both growing seasons using a split plot arrangement with a randomized complete plot design. Prior to analysis, the data were tested for distribution and variance homogeneity to ensure uniform error variance across the two growing seasons. The statistical program Costa (version 6.45, CoHort Software, VSN International Ltd., Oxford, UK) was used to conduct analysis of variance (ANOVA) and determine differences using the F-test. The post hoc test (Tukey’s test) at the 0.05 significance level was used to distinguish differences among the mean values. The heatmap clustering analysis was generated using R Studio software (version 2022.12.0+353, R Core Team, 2022) to visualize the integration of parameters across different treatments. Figures were created using Sigma Plot software (version 14.0; SPSS, Chicago, IL, USA).

## 5. Conclusions

This study highlights the importance of using an effective and complementary approach to improve wheat growth, productivity, and IWUE in arid regions under DI conditions. The exogenous foliar application of SA and nutrients has been found to be a successful approach for mitigating the negative effects of DI. Determining the best combinations of SA and nutrients as well as the timing of their application during key wheat growth stages is crucial for maximizing the benefits and enhancing wheat crop performance in arid regions with limited water resources. Our study showed that combining foliar SA with Mic nutrients enhanced wheat performance more effectively than using SA alone or in combination with both Mic and Mac nutrients. Additionally, we found that applying different components foliarly at only one early growth stage (tillering stage) did not provide any advantages. However, the foliar application of various components during both tillering and heading stages or during three stages (tillering, heading, and grain filling) resulted in the greatest benefits for enhancing wheat growth, production, and IWUE under DI conditions. In conclusion, this study suggests that applying SA and Mic foliarly at various growth stages can be a beneficial and supportive method to maintain wheat production under DI conditions. 

## Figures and Tables

**Table 1:** The analysis of variance (F-test) and mean comparisons of the effects of season (S), application timing (AT), the foliar treatments (F) of salicylic acid (SA), macronutrients (Mac), and micronutrients (Mic) and their interaction on different growth parameters of wheat at 95 and 115 days from sowing over two growing seasons.

Studied Factor		Growth Parameters at 95 Days from Sowing						Growth Parameters At 115 Days From Sowing					
		PH	TN	GLN	GLA	SFW	SDW	PH	TN	GLN	GLA	SFW	SDW
Application timing (AT)													
T		64.50 ^ns^	3.28 ^ns^	7.12 ^b^	96.63 ^b^	11.93 ^b^	3.86 ^b^	67.06 ^ns^	3.36 ^b^	2.74 ^b^	38.35 ^b^	10.38 ^b^	4.74 ^b^
T + H		65.39 ^ns^	3.40 ^ns^	8.22 ^a^	111.17 ^a^	13.54 ^a^	4.58 ^a^	67.71 ^ns^	3.65 ^a^	3.25 ^a^	44.96 ^a^	11.18 ^a^	5.58 ^a^
T + H + G		65.10 ^ns^	3.50 ^ns^	8.40 ^a^	113.40 ^a^	13.45 ^a^	4.60 ^a^	68.33 ^ns^	3.67 ^a^	3.34 ^a^	45.86 ^a^	11.46 ^a^	5.84 ^a^
Foliar treatments (F)													
Control		63.97 ^b^	3.26 ^ns^	7.07 ^b^	88.29 ^c^	11.43 ^c^	3.72 ^c^	65.69 ^b^	3.24 ^b^	2.56 ^b^	33.09 ^c^	9.07 ^c^	3.92 ^c^
SA		64.73 ^ab^	3.37 ^ns^	8.07 ^a^	113.54 ^ab^	13.44 ^ab^	4.57 ^ab^	68.00 ^a^	3.61 ^a^	3.36 ^a^	46.10 ^ab^	11.63 ^b^	5.91 ^a^
SA + Mic		65.70 ^a^	3.41 ^ns^	8.15 ^a^	116.41 ^a^	14.04 ^a^	4.76 ^a^	68.39 ^a^	3.67 ^a^	3.31^a^	48.08 ^a^	12.03 ^a^	6.06 ^a^
SA + Mic + Mac		65.58 ^a^	3.54 ^ns^	8.35 ^a^	110.03 ^b^	12.97 ^b^	4.31 ^b^	68.72 ^a^	3.72 ^a^	3.22 ^a^	44.96 ^b^	11.31 ^b^	5.64 ^b^
ANOVA	df												
S	1	0.109 ^ns^	0.421 ^ns^	0.502 ^ns^	0.336 ^ns^	0.723 ^ns^	0.053 ^ns^	0.333 ^ns^	0.185 ^ns^	0.103 ^ns^	0.601 ^ns^	0.526 ^ns^	0.063 ^ns^
AT	2	0.126 ^ns^	0.192 ^ns^	<0.001 ***	<0.001 ***	0.001 **	<0.001 ***	0.421 ^ns^	0.029 *	<0.001 ***	<0.001 ***	<0.001 ***	<0.001 ***
AT × S	2	0.372 ^ns^	0.491 ^ns^	0.031 *	0.468 ^ns^	0.582 ^ns^	0.682 ^ns^	0.692 ^ns^	0.009 **	0.594 ^ns^	0.974 ^ns^	0.187 ^ns^	0.058 ^ns^
F	3	0.006 **	0.259 ^ns^	<0.001 ***	<0.001 ***	<0.00 ***	<0.001 ***	0.001 **	0.001 **	<0.001 ***	<0.001 ***	<0.001 ***	<0.001 ***
F × S	3	0.533 ^ns^	0.644 ^ns^	0.537 ^ns^	0.150 ^ns^	0.999 ^ns^	0.704 ^ns^	0.367 ^ns^	0.775 ^ns^	0.624 ^ns^	0.870 ^ns^	0.276 ^ns^	0.119 ^ns^
F × AT	6	0.339 ^ns^	0.987 ^ns^	0.022 *	<0.001 ***	0.004 **	0.010 *	0.698 ^ns^	0.933 ^ns^	0.009 **	<0.001 ***	<0.001 ***	<0.001 ***
F × AT × S	6	0.333 ^ns^	0.528 ^ns^	0.563 ^ns^	0.506 ^ns^	0.991 ^ns^	0.899 ^ns^	0.463 ^ns^	0.750 ^ns^	0.580 ^ns^	0.231 ^ns^	0.701 ^ns^	0.236 ^ns^

Values are the means of three replications (n = 3). PH, plant height (cm plant^−1^); TN, tiller number per plant; GLN, green leaf number per plant; GLA, green leaves area (cm^2^ plant^−1^); SFW, shoot fresh weight (g plant^−1^), and SDW, shoot dry weight (g plant^−1^). T, T + H, and T + H + G indicate foliar application at tillering, tillering and heading, and tillering, heading, and grain filling growth stages, respectively. ns indicates non-significance while *, ** and *** indicate significance at *p* ≤ 0.05, 0.01 and 0.001, respectively, in F-tests. Means with the same letter within a column are not significantly different based on Tukey’s test.

**Table 2:** The analysis of variance (F-test) and mean comparisons of the effects of season (S), application timing (AT), the foliar treatments (F) of salicylic acid (SA), macronutrients (Mac), and micronutrients (Mic) and their interaction on different physiological parameters of wheat at 95 and 115 days from sowing over two growing seasons.

		Physiological Parameters at 95 Days from Sowing				Physiological Parameters at 115 Days from Sowing			
		RWC	Cha	Chb	Cht	RWC	Cha	Chb	Cht
Application timing (AT)									
T		67.11 ^b^	1.92 ^b^	0.79 ^b^	2.77 ^b^	57.72 ^b^	1.00 ^c^	0.44 ^c^	1.50 ^c^
T + H		75.26 ^a^	2.26 ^a^	0.93 ^a^	3.23 ^a^	62.72 ^a^	1.19 ^b^	0.51 ^b^	1.75 ^b^
T + H + G		76.50 ^a^	2.27 ^a^	0.91 ^a^	3.24 ^a^	63.85 ^a^	1.24 ^a^	0.53 ^a^	1.83 ^a^
Foliar treatments (F)									
Control		64.61 ^b^	1.47 ^b^	0.71 ^b^	2.22 ^b^	50.32 ^b^	0.74 ^c^	0.32 ^b^	1.10 ^c^
SA		75.86 ^a^	2.36 ^a^	0.93 ^a^	3.34 ^a^	64.53 ^a^	1.28 ^ab^	0.56 ^a^	1.90 ^ab^
SA + Mic		76.45 ^a^	2.40 ^a^	0.94 ^a^	3.39 ^a^	66.67 ^a^	1.32 ^a^	0.57 ^a^	1.94 ^a^
SA + Mic + Mac		74.90 ^a^	2.38 ^a^	0.93 ^a^	3.35 ^a^	64.20 ^a^	1.25 ^b^	0.54 ^a^	1.84 ^b^
ANOVA	df								
S	1	0.087 ^ns^	0.736 ^ns^	0.311 ^ns^	0.376 ^ns^	0.273 ^ns^	0.079 ^ns^	0.097 ^ns^	0.032 *
AT	2	<0.001 ***	<0.001 ***	<0.001 ***	<0.001 ***	0.001 **	<0.001 ***	<0.001 ***	<0.001 ***
AT × S	2	0.027 *	0.051 ^ns^	0.811 ^ns^	0.078 ^ns^	0.373 ^ns^	0.014 *	0.001 **	0.005 **
F	3	<0.001 ***	<0.001 ***	<0.001 ***	<0.001 ***	<0.001 ***	<0.001 ***	<0.001 ***	<0.001 ***
F × S	3	<0.001 ***	<0.001 ***	0.011 *	<0.001 ***	0.837 ^ns^	0.073 ^ns^	0.010 *	0.078 ^ns^
F × AT	6	<0.001 ***	<0.001 ***	<0.001 ***	<0.001 ***	<0.001 ***	<0.001 ***	<0.001***	<0.001 ***
F × AT × S	6	0.160 ^ns^	0.137 ^ns^	0.614 ^ns^	0.248 ^ns^	0.794 ^ns^	0.098 ^ns^	0.104 ^ns^	0.127 ^ns^

Values are the means of three replications (n = 3). RWC, Chla, Chlb, and Chlt indicate relative water content (%), chlorophyll a (mg g^−1^ FW), chlorophyll b (mg g^−1^ FW), and total chlorophyll content (mg g^−1^ FW), respectively. T, T + H, and T + H + G indicate foliar application at tillering, tillering and heading, and tillering, heading, and grain filling growth stages, respectively. ns, *, **, and *** indicate non-significant and significant at *p* ≤ 0.05, 0.01, and 0.001 in F-tests, respectively. Means with the same letter within a column are not significantly different based on Tukey’s test.

**Table 3:** The analysis of variance (F-test) and mean comparisons of the effects of season (S), application timing (AT), the foliar treatments (F) of salicylic acid (SA), macronutrients (Mac), and micronutrients (Mic) and their interaction on yield components, grain yield, and irrigation water use efficiency of wheat over two growing seasons.

		SL	GWPS	GNPS	TGW	GY	BY	HI	IWUE
		Application timing (AT)							
T		8.33 ^b^	1.05 ^c^	34.87 ^c^	30.23 ^b^	3.26 ^b^	11.08 ^b^	29.44 ^b^	9.86 ^b^
T + H		8.45 ^ab^	1.19 ^b^	36.86 ^b^	32.12 ^a^	3.74 ^a^	11.97 ^a^	31.16 ^a^	11.32 ^a^
T + H + G		8.62 ^a^	1.27 ^a^	38.90 ^a^	32.41 ^a^	3.87 ^a^	12.19 ^a^	31.65 ^a^	11.73 ^a^
		Foliar treatments (F)							
Control		8.33 ^ns^	0.94 ^d^	33.92 ^c^	27.96 ^d^	3.01 ^c^	10.67 ^b^	28.32 ^c^	9.14 ^c^
SA		8.44 ^ns^	1.25 ^b^	37.97 ^ab^	32.84 ^b^	3.88 ^a^	12.07 ^a^	32.06 ^a^	11.75 ^a^
SA + Mic		8.53 ^ns^	1.29 ^a^	38.37 ^a^	33.57 ^a^	4.01 ^a^	12.14 ^a^	32.98 ^a^	12.16 ^a^
SA + Mic+ Mac		8.58 ^ns^	1.19 ^c^	37.22 ^b^	31.99 ^c^	3.58 ^b^	12.09 ^a^	29.64 ^b^	10.83 ^b^
ANOVA	df								
S	1	0.383 ^ns^	0.802 ^ns^	0.704 ^ns^	0.913 ^ns^	0.051 ^ns^	0.007 **	0.306 ^ns^	0.051 ^ns^
AT	2	0.040 *	<0.001 ***	<0.001 ***	0.004 **	<0.001 ***	<0.001 ***	<0.003 **	<0.001 ***
AT × S	2	0.2.37 ^ns^	0.885 ^ns^	0.829 ^ns^	0.843 ^ns^	0.767 ^ns^	0.181 ^ns^	0.422 ^ns^	0.769 ^ns^
F	3	0.149 ^ns^	<0.001 ***	<0.001***	<0.001 ***	<0.001 ***	<0.001 ***	<0.001 ***	<0.001 ***
F × S	3	0.489 ^ns^	0.182 ^ns^	0.589 ^ns^	0.334 ^ns^	0.216 ^ns^	0.683 ^ns^	0.118 ^ns^	0.214 ^ns^
F × AT	6	0.748 ^ns^	<0.001 ***	<0.001 ***	<0.001 ***	<0.001 ***	<0.070 ^ns^	<0.001 ***	<0.001 ***
F × AT × S	6	0.051 ^ns^	0.982 ^ns^	0.697 ^ns^	0.731 ^ns^	0.980 ^ns^	0.985 ^ns^	0.799 ^ns^	0.980 ^ns^

Values are the means of three replications (n = 3). SL, spike length (cm); GNPS, grain number per spike; GWS, grain weight per spike (g); TGW, thousand-grain weight (g); GY, grain yield (ton ha^−1^); BY, biological yield (ton ha^−1^); HI, harvest index (%); IWUE, irrigation water use efficiency (kg mm^−1^ ha^−1^). T, T + H, and T + H + G indicate foliar application at tillering, tillering and heading, and tillering, heading, and grain filling growth stages, respectively. ns, *, **, and *** indicate non-significant and significant at *p* ≤ 0.05, 0.01, and 0.001 in F-tests, respectively. Means with the same letter within a column are not significantly different based on Tukey’s test.

## Data Availability

All data are presented in the article.
